# The synthetic self-hypothesis: dopaminergic redirection through self-face recognition in stuttering therapy

**DOI:** 10.3389/fnhum.2026.1824667

**Published:** 2026-06-16

**Authors:** Tolga Sözüçok

**Affiliations:** Department of Speech and Language Therapy, İzmir Tınaztepe University, İzmir, Türkiye

**Keywords:** AI therapy, dopamine, motor learning, reward-based learning, self-face recognition, stuttering

## Abstract

Stuttering affects approximately 1% of adults. Therapies based on imitation offer only modest benefits when individuals observe external models. Neurobiological research reveals a key asymmetry: some studies suggest self-face recognition activates ventral tegmental area (VTA) reward circuits more strongly than observing others’ faces, and positron emission tomography (PET) research shows increased dopamine synthesis in some people who stutter. The Synthetic Self Hypothesis (SSH) proposes that this increase reflects misdirected effort toward maladaptive targets rather than pathological excess. AI-generated fluent self-models may reroute dopaminergic reinforcement signals via two linked mechanisms: self-referential reward tagging, where VTA activation during self-face recognition boosts learning signals, and improved motor chunk consolidation, where phasic dopamine reinforcement strengthens fluent movement sequences. This framework leads to testable neural and behavioral predictions, including increased VTA–ventral striatum connectivity compared to VTA–dorsal striatum connectivity, larger reductions in stuttering frequency than waitlist controls, and outcomes mediated by increased communication self-efficacy. Baseline dopamine synthesis capacity is expected to influence treatment response, with those with higher synthesis capacity experiencing greater benefits from redirection-based therapies. In this view, the neurobiology of stuttering is considered a failure of dopamine targeting rather than dopamine pathology, offering a mechanistic basis for personalized AI-driven intervention while recognizing that metabolic constraints may set limits on treatment success.

## 1 Introduction

### 1.1 The problem of external modeling

Epidemiological research suggests that stuttering affects approximately 0.7% of the population across the lifespan (Craig et al., 2002). This disorder is characterized by involuntary disruptions in speech fluency, including word or syllable repetition, prolonged sounds, and speech blocks (Bloodstein and Bernstein Ratner, 2008). Among evidence-based interventions, speech restructuring treatments such as the Camperdown Program have demonstrated measurable reductions in stuttering severity through structured practice of modified speech patterns guided by clinician modeling and feedback (O'Brian et al., 2003; Cocomazzo et al., 2012). Such interventions usually entail learning a specified speech pattern designed to improve fluency. Despite significant improvements in fluency achievable through such therapeutic approaches, the extent of improvement varies from one patient to another.

A fundamental therapeutic question has received little attention in the stuttering literature: why should individuals who stutter imitate external models when the ultimate clinical goal is fluency in their own voice? This question becomes particularly relevant when considered in light of cognitive neuroscience evidence demonstrating that the brain processes self-related and externally related information through partially distinct neural systems.

Neuroimaging studies have consistently shown that observation of external faces activates the fusiform face area and posterior superior temporal sulcus, which are regions associated with general face perception (Haxby et al., 2000). Beyond these perceptual regions, observing one’s own face recruits additional structures, including the right inferior frontal gyrus and right inferior parietal lobule, which show increased specificity for self-related processing (Uddin et al., 2005). Importantly, self-face recognition has been *suggested* to engage the ventral tegmental area (VTA) reward circuits, a central component of the dopaminergic reinforcement system (Platek et al., 2008). This asymmetry suggests that self-related stimuli may receive enhanced neural prioritization through reward-related mechanisms. This raises the possibility that interventions leveraging self-recognition could produce stronger learning signals than those based solely on external modeling.

### 1.2 The dopamine paradox in stuttering

The relevance of this asymmetry becomes clearer when considered alongside findings regarding dopaminergic function in stuttering. Positron emission tomography studies using 6-[^18^F]fluoro-L-dopa (^18^F-FDOPA) have reported increased dopamine activity in a substantial subset of individuals who stutter, particularly within the cortical and limbic regions associated with speech production and motor control (Wu et al., 1997). These findings have contributed to the long-standing dopamine hypothesis of stuttering, which proposes that elevated dopaminergic signaling within cortico–basal ganglia circuits may play a role in the disorder (Maguire et al., 2002; Maguire et al., 2021). Although the precise neurochemical mechanisms remain under investigation, converging pharmacological and neuroimaging evidence suggests that dopaminergic modulation may be relevant for understanding the regulation of speech fluency in at least a subset of individuals who stutter.

Developmental timing further highlights the potential relevance of dopaminergic mechanisms. Dopamine plays a central role in cortico-striatal circuits involved in motor automatization and the internal generation of timing cues necessary for speech production (Alm, 2004; Alm, 2021a, 2021b). These systems undergo substantial maturation during childhood, the developmental period during which stuttering typically occurs (Wahlstrom et al., 2010). Persistent stuttering has been associated with alterations in cortico-basal ganglia networks (Chang et al., 2019; Rowe et al., 2024), suggesting that developmental changes in dopaminergic signaling may interact with the maturation of speech motor networks during the consolidation of fluent speech.

Current theoretical interpretations have largely framed dopaminergic alterations in stuttering as pathological. For example, elevated dopaminergic activity has been proposed to generate overly rigid motor programs through excessive policy precision (Alm, 2021b) or to contribute to aberrant precision weighting within predictive processing frameworks (Usler, 2025). However, this interpretation introduces a long-standing conceptual paradox. Dopamine functions as the brain’s primary reinforcement and learning signal, supporting adaptive behavior through reward-based learning mechanisms (Schultz et al., 1997; Friston et al., 2012). Why would the increased availability of a neurotransmitter central to learning and behavioral optimization lead to motor dysfunction?

This paradox suggests that the prevailing interpretations may be incomplete. We propose a reconceptualization: the critical issue may not be the quantity of dopamine but its targeting. In a subset of individuals who stutter, the elevated dopamine synthesis capacity observed in positron emission tomography (PET) studies may represent neural resources available for redirection rather than pathological excess requiring suppression (Wu et al., 1997; Alm, 2021b). Operationally, dopamine misdirection refers to the preferential reinforcement of maladaptive motor policies over adaptive ones, including rigid dorsal striatal habits, heightened sensory monitoring, and dysfluent motor routines, rather than flexible ventral striatal learning and consolidation of fluent motor chunks (Schultz, 2016). Such misdirection may be observable through differences in dorsal–ventral striatal connectivity or differential reinforcement dynamics for fluent versus dysfluent speech behaviors.

Under this account, the dopaminergic system itself operates normally in principle—it reinforces whichever behavioral patterns it accompanies. In typical speakers, successful communication reinforces fluent motor policies within the striatum and strengthens action-selection processes within cortico-striatal circuits (Schultz et al., 1997). However, in people who stutter, dopaminergic reinforcement may become biased toward behavioral patterns that rely heavily on habitual dorsal striatal control. Research on basal ganglia organization shows that the dorsal striatum plays a central role in the formation and stabilization of rigid motor habits and stimulus–response associations (Yin and Knowlton, 2006; Graybiel, 2008). When reinforcement learning processes disproportionately strengthen habitual control systems, behavioral patterns may become less flexible and more resistant to adaptive modification. Within this framework, elevated dopamine synthesis capacity could, in principle, coexist with impaired speech fluency if reinforcement processes preferentially stabilize maladaptive motor routines over flexible motor sequencing.

SSH does not propose a new account of stuttering onset. Persistent developmental stuttering involves genetic, developmental, and speech-motor factors that are well documented elsewhere (Frigerio-Domingues and Drayna, 2017; Kang et al., 2010). The Gradient Order Directions Into Velocities of Articulators (GODIVA) model (Bohland et al., 2010) provides a useful framework for understanding how dysfluent patterns may become reinforced: when syllable motor program selection is delayed, both elevated dopamine and white matter impairment can disrupt sequencing (Civier et al., 2013; Sommer et al., 2002). Therefore, SSH may target a possible mechanism of persistence rather than the developmental origins of the disorder.

### 1.3 The synthetic self-solution

Rather than attempting to reduce dopaminergic activity globally through medication or other interventions, therapeutic approaches may redirect existing dopamine toward more adaptive behavioral targets. The Synthetic Self Hypothesis proposes that AI-generated fluent self-models may achieve this redirection by exploiting the brain’s intrinsic reward response to self-related information (Oikawa et al., 2012; Ota and Nakano, 2021) in combination with the observation of fluent speech behavior. This process leverages the established self-face recognition network (Platek et al., 2008; Uddin et al., 2005) to ensure that the observed fluency is processed as a self-relevant, intrinsically rewarding signal rather than as an external motor template.

When individuals who stutter observe high-fidelity audiovisual representations of themselves speaking fluently, self-recognition processes engage neural systems associated with identity and personal relevance while simultaneously presenting fluent speech patterns. Converging evidence indicates that self-related processing can engage the reward circuitry, including the ventral striatum, ventromedial prefrontal cortex (vmPFC), and, under specific conditions, the ventral tegmental area (VTA) (de Greck et al., 2008; Haber and Knutson, 2010; Ota and Nakano, 2021). Through this mechanism, dopaminergic reinforcement may become more strongly associated with fluent motor patterns and fluent self-representations than dysfluent ones, thereby increasing the probability of reinforcing adaptive speech motor policies over maladaptive routines.

This approach differs critically from external modeling, in which the learning target is based on an external template that lacks personal identity significance. While observing others engages the mirror-neuron system for action understanding (Rizzolatti and Craighero, 2004; Rizzolatti et al., 1996), external models do not recruit specialized self-recognition networks that provide intrinsic reward signals (Uddin et al., 2005; Platek et al., 2008).

The Synthetic Self solution also differs from traditional video self-modeling (VSM) approaches, which are often constrained by the limited availability of naturally occurring fluent speech episodes (Cream et al., 2009, 2010). Artificial intelligence (AI) synthesis overcomes these limitations by enabling the controlled manipulation of fluency while preserving essential personal identity cues. This allows for both scalable therapeutic exposure and precise calibration of the relationship between a user’s current performance and their modeled fluency.

The theoretical foundation of this hypothesis integrates converging evidence from dopaminergic motor learning (Alm, 2021a, 2021b), active inference accounting for stuttering (Usler, 2025), and research on self-referential neural processing (Platek et al., 2008; Uddin et al., 2005; Northoff and Hayes, 2011). The following sections develop how they converge on two proposed mechanisms: self-referential reward tagging and dopamine-mediated motor chunk consolidation.

## 2 Theoretical framework: two core mechanisms

The Synthetic Self Hypothesis proposes a therapeutic benefit through two coupled neural mechanisms that together enable dopaminergic redirection. A third mechanism, involving action observation networks, may provide additional amplification; however, it is not unique to self-models. Therefore, the two core mechanisms are conceptualized as necessary components of the proposed intervention, interacting through reciprocal cortical–subcortical loops.

### 2.1 Core mechanism 1: self-referential reward tagging of learning signals

The first mechanism involves the self-referential reward tagging of learning signals. Self-face recognition recruits the right inferior frontal gyrus and right inferior parietal lobule with greater specificity than external face observation (Uddin et al., 2005). These regions support self–other discrimination processes that generate highly confident identity representations (Platek et al., 2008). Importantly, this self-recognition network interacts functionally with the ventral tegmental area (VTA) dopaminergic systems, linking identity processing with reward signaling (Northoff and Hayes, 2011).

When individuals who stutter observe AI-generated fluent self-videos, it is proposed that perceptual features would strongly signal self-recognition (for a detailed description of stimulus generation and quality control procedures, see section 4). Simultaneously, the observed fluent speech behavior contradicts the long-established expectations shaped by repeated experiences of dysfluency. This discrepancy generates a self-referential prediction error that may promote the updating of speech-related self-representations (Friston et al., 2012). In contrast, observing an external fluent speaker carries the cognitive content “that person is fluent” and does not directly challenge the observer’s own speech-related self-model (Rizzolatti et al., 1996; Platek et al., 2008).

The effective engagement of this mechanism depends on the critical window of fidelity. If synthetic videos diverge too far from an individual’s appearance or voice, self-recognition may fail. The stimulus may be processed as “not me,” consistent with contemporary findings on the uncanny valley effect in embodied conversational agents (Cihodaru-Ștefanache and Podina, 2025) and Bayesian explanations of perceptual distortion arising from conflicting cues (Moore, 2012).

Conversely, representations perceived as unrealistically fluent or “too human” to be authentic may also elicit rejection, as the pursuit of extreme realism can sometimes undermine the model’s epistemic foundation (Zeng et al., 2026). Optimal engagement is therefore expected when the synthetic model remains clearly recognizable as the self while presenting modest but attainable improvements in fluency. Such stimuli are likely to generate moderate prediction errors that are sufficient to motivate the updating of self-schemas without triggering perceptual rejection (Northoff and Hayes, 2011).

A related challenge may arise in cases of severe stuttering. Individuals with limited prior experience of fluent self-production may initially perceive moderate improvement as implausible. For these individuals, graduated calibration procedures may be required, in which early synthetic models display only minimal fluency improvements that progressively increase across sessions. This gradual calibration may allow the self-recognition window to expand over time rather than exceed the perceptual tolerance thresholds.

### 2.2 Core mechanism 2: phasic dopamine as a learning signal for motor automatization

The second core mechanism concerns phasic dopamine as a teaching signal for motor automatization. Motor learning is thought to involve chunking processes in which individual articulatory gestures are integrated into higher-order movement sequences (Alm, 2021b). Phasic dopamine release from ventral tegmental area (VTA) projections to the striatum—particularly the putamen—provides a reinforcement signal that supports the consolidation of such motor sequences within basal ganglia learning circuits (Schultz et al., 1997; Alm, 2021b).

We propose that in stuttering, this dopaminergic learning system may operate according to the same general principles but become directed toward less adaptive behavioral targets. In typical speakers, fluent articulatory patterns and self-representations may be reinforced through successful communicative outcomes (Schultz et al., 1997). However, in individuals who stutter, dysfluent motor patterns and heightened self-monitoring may receive inadvertent reinforcement through their behavioral consequences, including short-term anxiety reduction associated with speech avoidance (Menzies et al., 1999).

Consistent with the involvement of affective processes in speech, neuroimaging evidence from an interpersonal communication paradigm indicates that right amygdala activity during live interactions covaries with the occurrence of moment-to-moment stuttering (*r* = 0.54) and is associated with subjective discomfort during gazing (Toyomura et al., 2018). These findings suggest that socially evoked affective states may be coupled with disfluency during ongoing speech, thereby providing a potential pathway through which maladaptive reinforcement loops can be maintained.

Synthetic self-videos offer a mechanism for redirecting this reinforcement process. By pairing self-recognition with the observation of fluent speech behavior, the intervention may promote dopaminergic reinforcement signals that are temporally aligned with fluent motor representations rather than dysfluent ones. Repeated observation cycles may therefore create learning opportunities in which self-recognition triggers reward-related activity, while fluent articulatory patterns are simultaneously represented within the motor system (Alm, 2021b; Friston et al., 2012).

This mechanism depends on phasic rather than tonic dopamine signaling. Elevated tonic dopamine—reported in some individuals who stutter—may reduce sensitivity to phasic reinforcement signals by decreasing the signal-to-noise ratio of reward-related activity. This mechanism, originally proposed by Grace (1991) to explain dopaminergic dysregulation in other contexts, provides a principled basis for understanding how chronic baseline elevation can mask the precise timing of phasic learning signals during stuttering. Within this framework, the self-recognition response may function as a temporally precise trigger that can generate phasic reinforcement events, even in the presence of elevated tonic baseline levels.

The direct measurement of phasic dopamine in humans remains technically challenging. PET imaging integrates dopaminergic activity over relatively long time windows, while functional magnetic resonance imaging (fMRI) blood oxygen level-dependent (BOLD) responses reflect downstream hemodynamic changes rather than neurotransmitter release itself. Therefore, empirical evaluation of the mechanism will likely rely on convergent indicators of dopaminergic learning dynamics, including behavioral learning-rate modeling, physiological arousal indices such as pupillometry during stimulus exposure, and neuroimaging measures of functional connectivity within mesostriatal reward circuits. While indirect, these convergent measures allow rigorous testing of the proposed reinforcement-learning mechanism. This approach is motivated by evidence that experimentally induced discomfort and arousal during live gaze contexts track limbic responses, providing a principled rationale for including an arousal or engagement channel without claiming dopamine specificity (Toyomura et al., 2018).

### 2.3 Supporting mechanism: action observation amplification

Observation of speech actions engages the neural systems involved in both action perception and production. Research on action observations has shown that goal-directed actions recruit a network that includes the inferior frontal and premotor regions, which are associated with motor planning and execution (Rizzolatti and Craighero, 2004). Neuroimaging studies in humans have further indicated that the inferior frontal and premotor regions are activated when individuals observe or imitate others’ actions (Molenberghs et al., 2009). Consistent with this framework, observation and imitation of facial actions have also been shown to recruit the inferior frontal and premotor areas involved in action representation (Carr et al., 2003). Together, these findings suggest that action observations can engage motor-related representations that are also involved in action execution.

Neuroimaging and meta-analytic evidence suggest that distributed sensorimotor networks supporting speech production may play specific, dynamic, and context-dependent roles in speech perception, extending beyond classical motor regions (Skipper et al., 2017). Motor-system engagement during the observation of fluent speech is therefore not treated as a necessary decoder of speech perception but as a possible form of sensorimotor coupling that may facilitate prediction, imitation, and motor learning.

Within this framework, the observation of fluent speech may activate motor representations related to speech production. Repeated exposure to fluent articulatory patterns may therefore support sensorimotor recalibration within speech networks through mechanisms of experience-dependent plasticity (Pascual-Leone et al., 2005). Consistent with this possibility, neuroimaging studies have shown that behavioral fluency interventions can alter activation patterns within frontal speech regions and shift activity toward a more typical left-hemispheric organization (Neumann et al., 2003, 2005).

This mechanism should operate when observing both other speakers and one’s own speech behavior. However, we hypothesize that self-referential processing may amplify the motivational relevance of observed fluent speech when the observed speaker is the self. Specifically, self-recognition may increase the salience of observed motor patterns through interactions between reward-related systems and speech motor regions. Such salience modulation may be particularly relevant given the role of the basal ganglia and dopaminergic learning mechanisms in the timing and initiation of speech motor sequences (Civier et al., 2013).

Under this account, the magnitude of activation within the left inferior frontal regions may be comparable during observation of fluent speech produced by others and during observation of a synthetic fluent self-model. This critical difference between these two conditions is predicted to emerge at the network interaction level. When observing a fluent synthetic self-model, reward-related systems may increase the motivational relevance of the observed motor pattern, potentially strengthening the functional coupling between reward-related circuits and speech motor regions.

### 2.4 Integration with active inference: precision dynamics as context

Active inference theory provides a computational framework for understanding predictive processing in the brain (Friston, 2014; Friston and Frith, 2015). Within this framework, the brain maintains hierarchical generative models of the world and continuously updates these models by minimizing the prediction errors between the expected and incoming sensory signals. This process enables an organism to infer the hidden causes of its sensory inputs.

Precision weighting plays a central role in this process. Precision determines the relative influence of prediction errors during model updating and effectively regulates the balance between prior expectations and incoming sensory evidence. According to active inference accounts, dopamine modulates precision by regulating the gain assigned to prediction errors during belief updating (Friston, 2014).

Usler (2025) proposes that stuttering may reflect aberrant dynamics of precision. In this account, sensory precision remains excessively high in contexts where prior precision should dominate, preventing the sensory attenuation normally required for smooth speech production. Consequently, bottom-up sensory signals may overwhelm predictive control mechanisms, contributing to unstable speech motor execution.

Within this framework, the Synthetic Self Hypothesis proposes that exposure to fluent self-representations may strengthen fluency-related priors within predictive hierarchies. From a predictive processing perspective, stronger priors reduce reliance on moment-to-moment sensory feedback and increase the system’s tolerance for transient sensory fluctuations during speech production.

Predictive processing models further emphasize that hierarchical inference depends on generative models that link sensory inputs to their underlying causes. Within this framework, neural connectivity can be understood as encoding the causal structure of these generative models, allowing the brain to infer hidden states that give rise to sensory signals (Friston, 2012).

Taken together, the Synthetic Self Hypothesis situates the reinforcement of fluent self-representations within an active inference account of predictive regulation in speech production.

## 3 Testable predictions and falsifiability

### 3.1 Operationalization (primary endpoint)

To enable empirical testing, we operationalized the proposed dopaminergic redirection mechanism as a single network-level endpoint: a post-intervention increase in functional coupling between the ventral tegmental area (VTA) and ventral striatum relative to coupling between the VTA and dorsal striatum. This is quantified as the connectivity ratio [(VTA–ventral coupling)/(VTA–dorsal coupling)], where the coupling strength is estimated using Fisher z-transformed correlation coefficients derived from resting-state or task-based fMRI time series. In practical terms, successful redirection corresponds to a post-intervention increase in this ratio following exposure to Synthetic Self stimuli.

Support for this hypothesis requires that ventral striatal re-coupling emerges selectively in the Synthetic Self condition and correlates with measurable behavioral improvements in fluency. If functional connectivity within the VTA–ventral striatum pathway does not increase—or increases no more than the connectivity between the VTA and dorsal striatum—the proposed dopaminergic redirection mechanism is not supported. Additionally, if ventral striatal re-coupling occurs in the absence of corresponding behavioral improvement, this would indicate that connectivity changes alone are insufficient and that other factors (e.g., metabolic constraints, insufficient training exposure, or individual baseline differences) moderate the translation from neural reorganization to functional outcomes.

Other indicators of dopaminergic learning dynamics are treated as convergent; however, secondary evidence validates the primary connectivity-based mechanism. These may include PET-based dopamine synthesis indices, pupillometric markers of arousal during stimulus exposure, and behavioral learning-rate modeling derived from reinforcement learning frameworks. While these measures cannot directly index phasic dopamine release in humans, they provide indirect yet informative indicators of dopaminergic system engagement.

Predictions are organized into tiers, reflecting both mechanistic importance and empirical accessibility. Tier 1 predictions are hypothesis-critical; null findings at this level would require substantial revision or rejection of the proposed mechanism. Tier 2 predictions provide supporting evidence for the framework but are not individually decisive for evaluating the central hypothesis.

### 3.2 Tier 1: hypothesis-critical predictions

#### 3.2.1 Prediction 1: ventral striatal re-coupling

Relative to control conditions, exposure to Synthetic Self stimuli will produce a post-intervention increase in functional coupling between the ventral tegmental area (VTA) and ventral striatum, compared with coupling between the VTA and dorsal striatum. Quantitatively, this corresponds to an increase in the connectivity ratio [(VTA–ventral coupling)/(VTA–dorsal coupling)], where the coupling strength is estimated using Fisher z-transformed correlation coefficients derived from fMRI time series.

In other words, the VTA–ventral/VTA–dorsal striatal connectivity ratio is expected to shift toward ventral striatal dominance following the intervention. If this connectivity shift does not occur—or occurs equally across experimental and control conditions—the proposed dopaminergic redirection mechanism is not supported. Additionally, if ventral striatal re-coupling occurs without corresponding behavioral improvement, this would indicate that connectivity changes are necessary but insufficient, suggesting that other factors (e.g., metabolic constraints, training dosage, or baseline severity) moderate the translation from neural reorganization to functional outcomes.

#### 3.2.2 Prediction 2: superiority in stuttering reduction

Participants exposed to the Synthetic Self intervention will show significantly greater reductions in stuttering frequency than participants in the waitlist control condition. A measurable improvement in fluency is expected relative to baseline performance, reflecting the successful stabilization of speech motor chunks. Clinically meaningful improvement will be operationalized as a significant and sustained reduction in percentages of syllables stuttered (%SS), aligning with established clinical outcome standards for adult stuttering interventions (O’Brian et al., 2024; Harasym et al., 2015). If the Synthetic Self condition fails to produce reductions exceeding those observed in the waitlist control in an adequately powered study, the primary clinical claim of the hypothesis would be falsified.

#### 3.2.3 Prediction 3: self-efficacy mediation

Changes in communication self-efficacy, measured using established instruments such as the Self-Efficacy Scale for Adult Stutterers (Ornstein and Manning, 1985), partially mediate the relationship between intervention exposure and stuttering reduction. Improvements in self-efficacy are expected to exert a statistically significant indirect effect on fluency outcomes in mediation analyses. Prior work indicates that self-efficacy is strongly associated with quality of life and stuttering severity in adults who stutter (Carter et al., 2017).

If no such indirect effect is observed, the proposed identity-transformation mechanism would not be supported, suggesting that any observed fluency improvements arise from alternative mechanisms, such as motor practice or exposure-related habituation.

### 3.3 Tier 2: supporting predictions

#### 3.3.1 Prediction 4: inverted-U self-resemblance function

VTA activation during synthetic self-observation is expected to vary as a function of perceived self-resemblance. Parametric fMRI analysis will test whether reward-related activation increases as synthetic faces approach the participant’s own facial identity and decreases when resemblance is either too low or perceptually implausible.

This prediction draws on the morph-continuum paradigms used in self-recognition research, where faces containing varying proportions of self and other identity systematically alter self-recognition judgments (Turk et al., 2002). Within this framework, Synthetic Self stimuli that fall within an optimal resemblance range may produce the strongest reward-related responses, whereas faces that are insufficiently self-like or perceptually distorted may fail to engage in self-referential reward processing.

Testing this gradient allows empirical evaluation of the proposed fidelity window as well as potential perceptual rejection effects consistent with the uncanny valley phenomenon.

#### 3.3.2 Prediction 5: neural–behavioral coupling

The magnitude of VTA activation during exposure to synthetic self-videos is expected to positively correlate with improvements in stuttering frequency across participants. Individuals showing stronger reward-system engagement during self-observation should demonstrate larger reductions in percent syllables stuttered (%SS) following the intervention. Evidence of such a relationship would indicate neural–behavioral coupling between reward-system activation and therapeutic outcomes.

#### 3.3.3 Prediction 6: moderation by dopamine synthesis capacity

Baseline dopamine synthesis capacity, assessed using ^18^F-FDOPA PET, will moderate treatment response. Individuals with moderate to high baseline dopamine synthesis capacity in the VTA and striatal regions are expected to exhibit greater improvements in stuttering frequency than individuals with low synthesis capacity. This prediction operationalizes the dopamine availability hypothesis and provides a basis for personalized treatment selection.

Taken together, these predictions outline a mechanistic pathway in which self-face recognition engages dopaminergic reward circuits in the VTA, reinforcing fluent speech motor patterns through reward-based learning.

### 3.4 Competing hypotheses and discriminating tests

Several alternative explanations should be considered when evaluating the proposed mechanism. If improvements arise primarily from expectancy effects rather than dopaminergic redirection, the Synthetic Self condition should show no advantage over a Sham AI control condition in which participants are told they are viewing an “AI-processed version of themselves” but are in fact shown their own unmodified recordings. This manipulation controls expectancy and self-exposure without synthetic fluency enhancement. Under this expectancy account, both conditions should produce comparable neural and behavioral outcomes. In contrast, the Synthetic Self Hypothesis predicts that true self-model stimuli will produce stronger VTA activation and greater reductions in stuttering frequency than in the Sham AI condition.

A second competing explanation involves exposure-based anxiety habituation (Menzies et al., 1999). According to this account, repeated exposure to fluent speech models may reduce speech-related anxiety and improve fluency, regardless of model identity. If this mechanism alone explains the improvement, Synthetic Self and External Fluent Model conditions should produce similar outcomes. The Synthetic Self Hypothesis predicts superior outcomes in the Synthetic Self condition despite equivalent exposure durations, indicating that self-referential reward mechanisms contribute to outcomes beyond simple habituation.

## 4 Intervention operationalization

Synthetic self-models are generated using generative artificial intelligence architectures rather than selective editing of previously recorded fluent segments. The procedure begins by collecting approximately 15–20 min of participant speech to establish a high-fidelity neural representation of the participant’s vocal and facial characteristics. These recordings are used to construct individualized voice and facial identity models, which serve as the basis for generative synthesis.

Audio-to-video diffusion architectures then generate fluent speech sequences that preserve the participant’s acoustic and facial identity while reconstructing speech behavior that the participant had not previously produced. In contrast to conventional video self-modeling approaches—which rely on editing previously recorded fluent moments—the present system produces counterfactual fluent self-representations, enabling the generation of stable, coherent fluent speech performances that go beyond fidelity-based editing of existing behavior. The resulting stimulus, therefore, represents a plausible fluent version of the speaker rather than a replay of past performance.

Quality control procedures ensure that the generated stimuli remain perceptually recognizable to the participant. Participants rate the perceived self-resemblance of each generated video on a 10-point scale, and stimuli are accepted only if the resemblance rating is ≥ 7/10. This criterion operationalizes the proposed *fidelity window*, ensuring that the stimulus remains sufficiently self-like to engage in self-referential processing while avoiding perceptual estrangement.

For participants with severe stuttering, a graduated calibration procedure is implemented. Fluency enhancement is gradually introduced by systematically reducing the probability of synthesized dysfluency across sessions. Progression occurs every three sessions contingent upon two conditions: (i) perceived self-resemblance ratings remain ≥7/10, and (ii) participants report no subjective sense of self-estrangement or identity mismatch. This calibration procedure is designed to maintain perceptual plausibility while progressively increasing the fluency of the synthetic self-model.

The intervention follows a 12-week protocol. Week 1 is devoted to baseline assessment and stimulus synthesis. Weeks 2 through 8 involve intensive daily exposure sessions of approximately 20 min. Weeks 9 through 12 constitute a maintenance phase emphasizing the generalization of speech patterns to real-world communication contexts.

The session structure follows the established principles of motor learning and skill acquisition. Each session includes three components. The first phase is an encoding phase (6 min), during which participants observe the fluent synthetic self-model. The second phase is a consolidation phase (10 min), in which participants imitate and compare their own productions with the model. The third phase is a free practice phase (4 min), during which participants engage in unguided speech production tasks.

The overall 20-min session duration is informed by motor learning research, suggesting that repeated short practice blocks can engage dopamine-dependent mechanisms of motor plasticity (Hosp and Luft, 2013). Intersession intervals of approximately 24 h are used to facilitate sleep-dependent consolidation processes that are known to support motor learning and skill stabilization (Walker et al., 2002). Speech motor chunk formation may also depend on phasic dopaminergic reinforcement during comparable learning processes (Alm, 2021b).

## 5 Design and methodology

The study employs a four-arm randomized design consisting of four experimental conditions: Synthetic Self intervention, Camperdown speech restructuring therapy with clinician modeling, Generic Fluent Speaker condition, and Sham AI condition. This design is intended to separate the effects of generative self-modeling from standard therapy effects, general exposure to fluent speech models, and expectancy-driven improvements.

To address expectancy and placebo-like effects, the proposed design includes both passive and active control functions. A waitlist control may be retained to estimate changes associated with time, repeated assessment, and spontaneous fluctuation. However, because exposure to an AI-based self-video intervention may generate expectancy effects, the design also includes a Sham AI condition in which participants view their own unmodified recordings described as “AI-processed.” This condition is intended to control for self-exposure, novelty, and expectancy without providing synthetic fluency enhancement.

In addition, the Synthetic Self condition is compared with an established standard intervention, the Camperdown Program, which represents a clinician-guided speech restructuring approach with documented clinical efficacy (O’Brian et al., 2024). This comparison allows evaluation of whether SSH produces outcomes beyond both expectancy/self-exposure effects and the currently available standard fluency intervention. Finally, the Generic Fluent Speaker condition controls for exposure to fluent speech models without self-referential identity cues.

The primary outcome measure is percent syllables stuttered (%SS), assessed at baseline, 4 weeks, 8 weeks, 12 weeks, and at a 6-month follow-up. All speech samples will be scored blindly according to the experimental condition.

Secondary outcomes include communication self-efficacy, measured using the Self-Efficacy Scale for Adult Stutterers (Ornstein and Manning, 1985), and stuttering-related quality of life, assessed using the Overall Assessment of the Speaker’s Experience of Stuttering (OASES; Yaruss and Quesal, 2006). These measures align with contemporary clinical outcome frameworks that emphasize speech performance and participation-level outcomes.

### 5.1 Mechanism study

A Phase 2 mechanism study (*n* = 40) will investigate the neural processes underlying the proposed intervention. Participants will undergo midbrain-optimized functional magnetic resonance imaging targeting the activity within the ventral tegmental area (VTA).

Baseline ^18^F-FDOPA PET imaging will be conducted in a stratified subsample (*n* ≈ 16) representing the low-, medium-, and high-dopamine-synthesis groups. Given the small size and susceptibility artifacts associated with the VTA, imaging will employ high-resolution midbrain-optimized acquisition protocols with distortion correction and physiological noise modeling combined with *a priori* region-of-interest analyses to maximize sensitivity.

Functional connectivity analyses will examine mesostriatal reward circuitry, particularly the interactions between the VTA and striatal regions implicated in reinforcement learning.

Within this framework, observing a generatively produced fluent version of one’s own speech may engage neural systems involved in self-referential processing and episodic future simulations (Szpunar et al., 2007). Such self-relevant simulations may increase motivational salience compared with externally generated models. Enhanced salience may amplify reinforcement signals within mesostriatal dopaminergic pathways, biasing speech motor selection toward more stable articulatory patterns through reward-based learning mechanisms.

## 6 Limitations and moderators

Individual differences in baseline metabolism may moderate the treatment response, defining boundary conditions for SSH effectiveness. Alm (2021a) presents comprehensive evidence that neuronal energy supply limitations may contribute to stuttering in a subset of cases. This evidence includes reduced electroencephalography (EEG) beta power at rest across multiple studies, which correlates with metabolic activity levels; patterns of reduced regional cerebral blood flow in frontal regions that correspond to areas with high glycolytic index, indicating reliance on non-oxidative glucose metabolism; genetic variants in aryl hydrocarbon receptor nuclear translocator 2 (ARNT2) affecting cellular oxygen sensing and glycolysis activation; and lysosomal gene mutations in N-acetylglucosamine-1-phosphate transferase subunits alpha and beta (GNPTAB), N-acetylglucosamine-1-phosphate transferase subunit gamma (GNPTG), and N-acetylglucosamine-1-phosphodiester alpha-N-acetylglucosaminidase (NAGPA) that reduce cellular metabolic processing capacity. Mouse models carrying stuttering-associated lysosomal mutations exhibit reduced astrocyte numbers and prolonged pauses between vocalizations, which is consistent with energy supply constraints. Metabolic factors are not proposed as core mechanisms of the Synthetic Self Hypothesis, but as boundary conditions that may moderate individual responsiveness.

If significant energy limitations exist, the capacity to sustain SSH’s neurally demanding processes—including self-face processing, VTA activation, and motor simulation—may be compromised. Interestingly, Alm (2021a) noted that VTA dopamine activation can increase cerebral glucose consumption and modulate astrocytic glycogen metabolism, suggesting potential compensatory mechanisms. However, whether VTA activation through SSH provides sufficient metabolic support in energy-limited individuals remains unclear.

We propose that SSH effectiveness is optimal in individuals with normal-to-elevated baseline dopamine synthesis capacity measurable via 18F-FDOPA PET and in the absence of metabolic genetic variants. Alm (2021a) advocated parallel exploration of metabolic and dopaminergic mechanisms. Accordingly, baseline metabolic and dopaminergic screening may enable stratified treatment selection and identification of optimal candidates for dopaminergic redirection compared with those requiring metabolic intervention as a primary or adjunctive approach.

Current AI technology also has practical limitations, requiring quality thresholds and iterative refinement (Moore, 2012). Photorealistic artifacts, prosodic naturalness, and emotional authenticity may affect self-resemblance ratings and therapeutic engagement. Self-estrangement risk requires active management through gradual divergence progression, continuous monitoring with weekly self-resemblance ratings, and participant control. Ethical safeguards include informed consent to address AI and deepfake risks, encrypted data storage, watermarking to prevent misuse, neurodiversity framing of fluency as an option rather than an obligation, and vulnerability screening to exclude individuals with body dysmorphic disorders, dissociative conditions, or active psychosis.

## 7 Research program and future directions

An empirical evaluation requires a phased approach. Phase 1 proof-of-concept with 15–20 participants establishes the feasibility, acceptability, safety, and preliminary efficacy of AI generation. A Phase 2 mechanism study with 40 participants investigates VTA activation using within-subject neuroimaging designs that compare synthetic self-versus external models, with midbrain-optimized fMRI and PET convergent validation, includes a baseline 18F-FDOPA assessment to examine dopamine synthesis capacity as a moderator, and produces effect size estimates for Phase 3 power calculation. A Phase 3 randomized controlled trial with 120 participants compares four conditions, with primary, secondary, and tertiary outcomes, and a six-month follow-up; stratified analyses examine etiology, presentation type, baseline dopamine synthesis capacity, and severity as moderators.

This framework shifts the conceptualization of stuttering interventions from direct motor correction to reinforcement-driven recalibration of predictive speech control. Rather than attempting to suppress dysfluency solely through compensatory strategies, the Synthetic Self Hypothesis proposes that fluent self-representations may reshape speech-motor expectations through reward-mediated learning mechanisms.

## 8 Discussion

The preceding sections argued that dopaminergic alterations in stuttering may reflect a failure of targeting rather than a failure of supply. Building on this framework, several theoretical traditions converge on a common implication: self-referential reward signals may offer a principled route for redirecting reinforcement toward fluent motor representations. Alm’s work provides a neurobiological account of phasic dopamine in motor chunk consolidation and highlights the potential metabolic constraints on motor learning (Alm, 2021a, 2021b). Usler’s active inference model describes how altered precision dynamics may destabilize predictive control in speech production (Usler, 2025). Research on self-referential processing demonstrates that self-related stimuli recruit partially distinct neural networks compared to externally generated information (Platek et al., 2008; Uddin et al., 2005). The Synthetic Self Hypothesis brings these frameworks together by proposing that self-referential reward-tagging may redirect dopaminergic learning signals toward fluent speech representations (Friston and Frith, 2015; Alm, 2021b), while acknowledging that metabolic and neurobiological factors may set boundary conditions for intervention effectiveness.

The SSH framework aligns with neuroscientific research on episodic future thoughts (Szpunar et al., 2007). When individuals envision their personal future, neural networks—including the parahippocampal gyrus and posterior cingulate—reactivate visual–spatial contexts from past experiences to construct plausible future scenarios. By presenting a generative, fluent self-model, SSH can engage these future self-projection networks. This allows individuals who stutter to mentally simulate—and potentially consolidate through repeated exposure—a fluent future identity. This process complements dopaminergic reinforcement by establishing coherent prospective self-representations that support goal-directed motor learning.

Importantly, the framework generates specific testable predictions that allow rigorous empirical evaluation. If VTA activation does not differentiate synthetic self-models from external models across convergent measurement approaches, behavioral outcomes do not exceed those observed in control conditions, self-efficacy does not mediate treatment effects, or baseline dopamine synthesis capacity does not moderate outcomes, the central claims of the hypothesis would require revision. This emphasis on falsifiability distinguishes the proposal from post-hoc explanatory models that cannot be empirically evaluated.

Contemporary stuttering interventions increasingly emphasize acceptance-based approaches aligned with the International Classification of Functioning (ICF) framework, which prioritizes communicative participation over the elimination of speech impairment (Yaruss and Quesal, 2004). Interventions such as Acceptance and Commitment Therapy and Avoidance Reduction Therapy aim primarily to reduce anxiety-driven behavioral patterns rather than directly modifying motor control processes (Menzies et al., 2008, 2009; Beilby et al., 2012). The Synthetic Self Hypothesis is compatible with, and potentially complementary to, this perspective. Desensitization-based approaches may reduce amygdala-mediated interference with reward-based learning, creating a neurobiological context that enables dopaminergic reinforcement signals to consolidate fluent motor patterns more effectively. In this view, fluency is not positioned as an obligatory therapeutic goal but as an option that becomes accessible once anxiety no longer constrains learning processes. Therefore, improvements in self-efficacy and identity transformation may emerge, even in the absence of substantial behavioral change.

If empirically supported, this framework may have important clinical implications. Many current fluency interventions rely on the imitation of external models (O'Brian et al., 2003; O’Brian et al., 2024). Here, imitation-based or model-based approaches refer specifically to interventions in which fluency is supported through exposure to, imitation of, or comparison with an external speech model, including clinician-modeled speech restructuring programs such as the Camperdown Program, choral or imitated speech paradigms, and video self-modeling. This does not imply that all stuttering treatments are model-based. Stuttering modification approaches, including voluntary stuttering, may involve clinician modeling, but their primary aim is to reduce avoidance, fear, struggle, and loss of control during moments of stuttering, rather than to establish fluency through external fluent-speech modeling. Although such approaches engage speech motor systems, they may not strongly engage self-referential reward-processing mechanisms. This limitation may contribute to the modest treatment effects and relapse patterns reported for some fluency-based interventions. In contrast, synthetic self-models may simultaneously engage action observation networks involved in speech production (Rizzolatti and Craighero, 2004) and reward-based learning systems activated by self-recognition (Platek et al., 2008; Schultz et al., 1997). If this dual engagement strengthens the consolidation of fluent motor representations, the result may be more durable therapeutic effects in individuals with sufficient dopaminergic and metabolic capacity. In the long term, the baseline assessment of dopamine synthesis capacity and metabolic indicators may support more personalized treatment selection, allowing clinicians to match intervention strategies to individual neurobiological profiles.

The proposed dopaminergic redirection mechanism also bears a conceptual similarity to mindfulness-based approaches, although the two approaches may operate through different therapeutic pathways. Mindfulness-based interventions may influence reward-related and dopaminergic systems; for example, meditation has been associated with increased endogenous dopamine release in the ventral striatum (Kjaer et al., 2002), and mindfulness-based training has been linked to altered functional connectivity between interoceptive and reward-related regions (Fedeli et al., 2025). In stuttering interventions, mindfulness-based approaches have been proposed to support present-moment awareness, acceptance, and reduced reactivity to speech-related sensations and emotions (Plexico and Sandage, 2011). Mindfulness may act primarily through interoceptive awareness, emotional regulation, and the reduction of anxiety-driven interference during speech.

A related distinction can be drawn with Van Riper’s stuttering modification approach, which includes visual self-confrontation through mirrors, photographs, or videotapes as part of the identification and desensitization procedures (Van Riper, 1973). With this approach, clients may observe the visible articulatory and facial components of their stuttering and gradually tolerate the visual experience of dysfluent speech behavior. SSH differs from these approaches by presenting an externally generated, high-fidelity representation of fluent self-speech rather than directing attention primarily toward internal sensations or current dysfluent behavior. SSH also differs from suggestion-based approaches such as hypnosis in that the therapeutic effect is proposed to arise from externally generated perceptual evidence rather than internally generated imagery, potentially offering benefit to individuals for whom internal fluency simulation is constrained by entrenched dysfluency expectations.

These approaches should therefore be viewed as potentially complementary rather than competing. Mindfulness may enhance interoceptive awareness and reduce affective reactivity, Van Riper-style visual self-confrontation may support identification and desensitization to current dysfluent behavior, and SSH may provide perceptual evidence of alternative fluent motor outcomes that could support reward-mediated learning.

The present hypothesis emphasizes audiovisual rather than audio-only self-models because SSH is proposed to depend on the convergence of several self-referential and sensorimotor cues. Visual facial identity may support self-recognition, visible articulatory movements may provide motor-relevant information about fluent speech production, and auditory signals may preserve aspects of vocal identity and prosody. This audiovisual emphasis is consistent with action-observation approaches in aphasia rehabilitation, such as the Intensive Mouth Imitation and Talking for Aphasia Therapeutic Effects (IMITATE) protocol, in which patients observe audio-visually presented words and phrases prior to oral repetition (Small, 2009), and with mirror-neuron-based aphasia intervention research that demonstrates stronger sensorimotor network engagement during action observation than during static object observation (Chen et al., 2019).

The clinical relevance of visual articulatory information is further supported by research on choral speech and its derivatives for stuttering. Kalinowski and Saltuklaroglu (2003) proposed that choral speech may reduce stuttering through gestural imitation and mirror-system engagement. They also discussed evidence that observing another speaker silently mouthing linguistic material without any accompanying acoustic signal may substantially reduce the stuttering frequency. This suggests that visible articulatory gestures may carry fluency-relevant information, even in the absence of an acoustic signal, providing indirect empirical support for the audiovisual emphasis of SSH over audio-only alternatives.

Nevertheless, audio-only fluent self-models may have therapeutic potential and warrant direct empirical testing. Listening to one’s own fluent voice could theoretically engage self-voice recognition and auditory–motor prediction mechanisms. However, because audio-only stimuli do not provide facial identity or visible articulatory cues, they may engage the proposed SSH mechanism less strongly than multimodal audiovisual stimuli.

A further methodological consideration concerns recorded self-voice perception. During natural speaking, voice perception includes both air- and body-conducted components, whereas a recorded or synthetic self-voice primarily contains an air-conducted component. This acoustic mismatch may reduce the perceived familiarity, authenticity, or ownership of a recorded or synthetic self-voice. Therefore, future studies adopting the SSH framework should assess perceived voice resemblance alongside facial self-resemblance and directly compare audio-only and audiovisual self-model conditions to clarify these modality-specific constraints.

The Synthetic Self Hypothesis therefore proposes that fluent self-representations may function as high-salience reinforcement signals capable of reshaping speech motor expectations through reward-mediated learning processes. By integrating predictive processing, dopaminergic reinforcement, and self-referential neural systems, this framework offers a testable pathway through which emerging AI technologies may support new forms of neurobiologically informed interventions for stuttering.

## 9 Conclusion

The Synthetic Self Hypothesis represents a shift in how dopaminergic function in stuttering may be understood—not as a pathological excess requiring suppression, but as a redirectable resource. AI-generated fluent self-models offer a theoretically motivated mechanism for leveraging the self-referential reward circuitry to bias reinforcement toward fluent motor representations. Whether this mechanism translates into durable clinical benefits remains an open empirical question; however, the hypothesis is sufficiently specified to permit rigorous testing.

This hypothesis further suggests that individual responses to treatment may vary according to baseline neurobiological resources, including dopamine production capacity and other metabolic factors. Such individual differences may significantly influence which participants derive the greatest benefit from the intervention.

## Data Availability

The original contributions presented in the study are included in the article/supplementary material, further inquiries can be directed to the corresponding author.
